# A case of varicella due to primary varicella zoster virus infection followed by respiratory disease on the background of an immunocompromised condition

**DOI:** 10.1002/ccr3.8422

**Published:** 2024-01-08

**Authors:** Hizuru Tomita, Yoshimasa Nobeyama, Miya Morishima, Akihiko Asahina

**Affiliations:** ^1^ Department of Dermatology The Jikei University School of Medicine Tokyo Japan

**Keywords:** cytomegalovirus activation, immunocompromised condition, systemic lupus erythematosus, varicella, varicella zoster virus infection

## Abstract

We encountered an immunocompromised patient with severe respiratory disease immediately after the onset of varicella. Varicella zoster virus infection may be associated with more severe immunosuppressive condition.

## Graphical abstract

Clinical findings of varicella which preceded CMV reactivation and pneumonia. (A) Lower power magnification; (B) High power magnification.

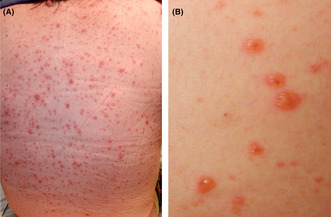

## 1 INTRODUCTION

Varicella zoster virus (VZV) infection potentially leads to serious outcomes especially in immunocompromised patients. VZV infection is reported to induce apoptosis of lymphocytes, resulting in decrease of lymphocyte count in the peripheral blood.^1^, ^2^ In that context, special caution may be needed after the onset of VZV‐infectious disease especially for immunocompromised patients. We encountered an immunocompromised patient with the onset of varicella as primary VZV infection immediately followed by severe respiratory disease.

## 2 CASE REPORT

**FIGURE 1:**
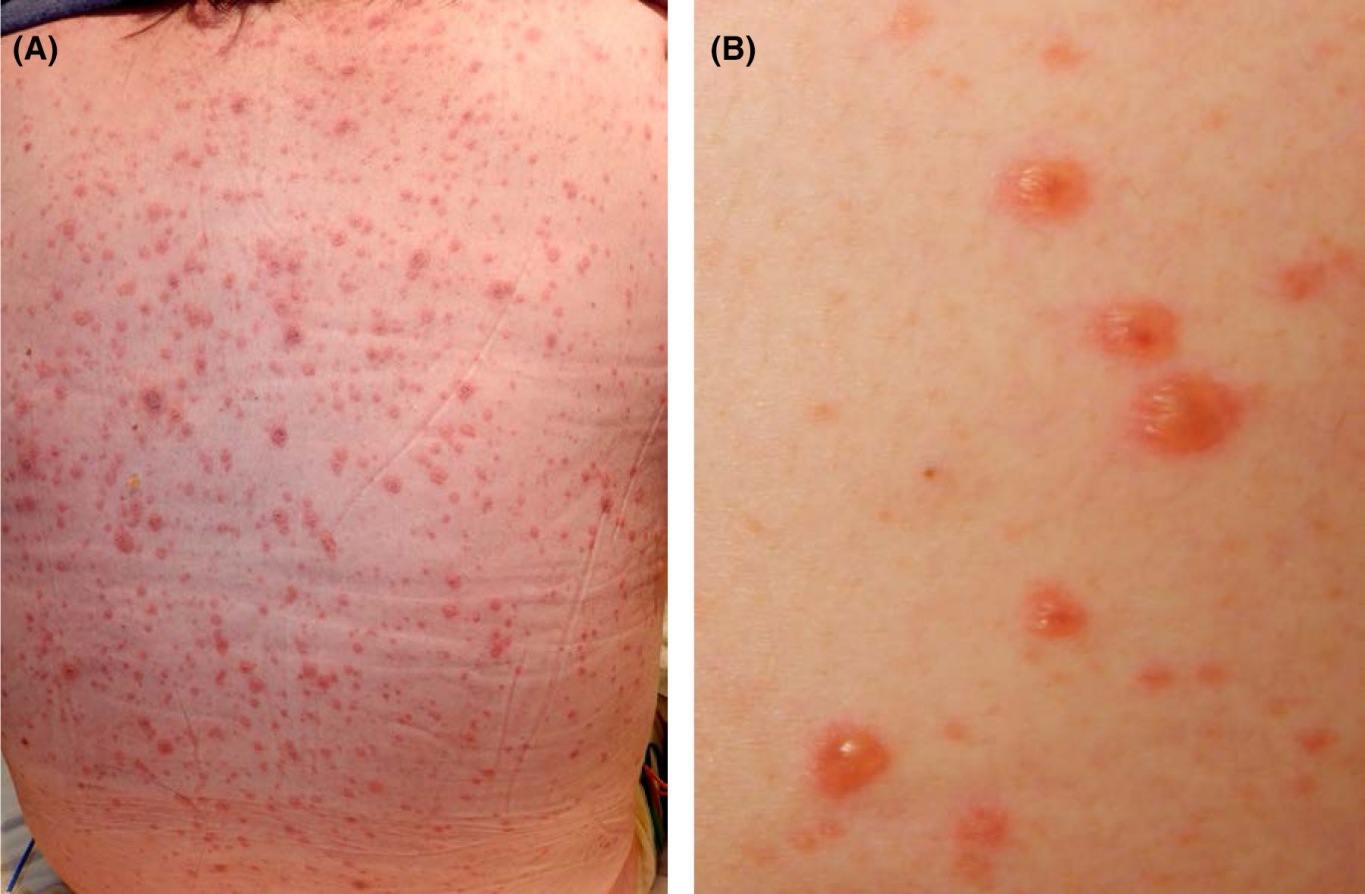
Clinical manifestations in the first visit. (A) Papules, papular vesicles, crusted vesicles, and crusts are mutually intermingled on the back. (B) Vesicles <6 mm in diameter are shown. Some vesicles have an umbilication representing necrosis in the center.

**FIGURE 2:**
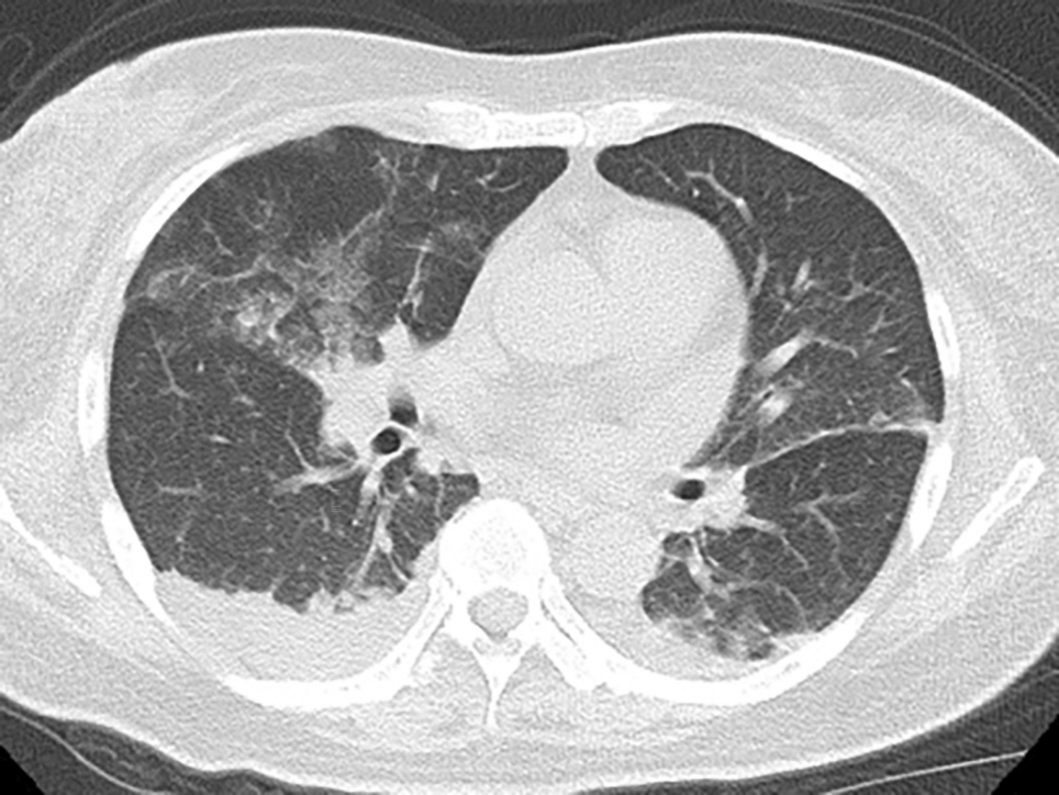
CT imaging of lungs. Ground‐glass opacities are observed in the bilateral lungs; however, no nodules suggestive of varicella pneumonia are found.

A 49‐year‐old female was referred to us with a 4‐day history of cutaneous manifestations on the trunk and extremities. As a past medical history, it was not certain whether the patient received a vaccination against VZV and experienced varicella. The patient had been diagnosed with systemic lupus erythematosus (SLE) 2 months earlier according to the European League Against Rheumatism and the American College of Rheumatology (2019) criteria^3^ and remained hospitalized. SLE was controlled well by a treatment with prednisolone at 0.7 mg/kg/day, mycophenolate mofetil at 2000 mg/day, and hydroxychloroquine at 200 and 400 mg on alternate days. Four days before presentation, the patient developed red papules on the right lower jaw. Two days before, dyspnea with pyrexia occurred and, thus, the administration of oxygen was initiated. In the first visit, the eruptions became vesicles, which spread over the body (Figure 1). The Tzanck test and immunochromatography to detect VZV antigens (DermaQuick VZV; Marho, Osaka, Japan) for vesicles were both positive. In blood tests, VZV‐specific IgM and VZV‐specific IgG examined using enzyme‐linked immunosorbent assays (ELISA) were 0.38 (negative range, <0.8) and <2.0 (negative range, <2.0), respectively, while a cytomegalovirus (CMV) antigen‐detecting test using the C7 horseradish peroxidase method was positive (four positive cells per 50,000 white blood cells). Bilateral ground‐glass opacities were observed on chest computed tomography (CT) (Figure 2).

**FIGURE 3:**
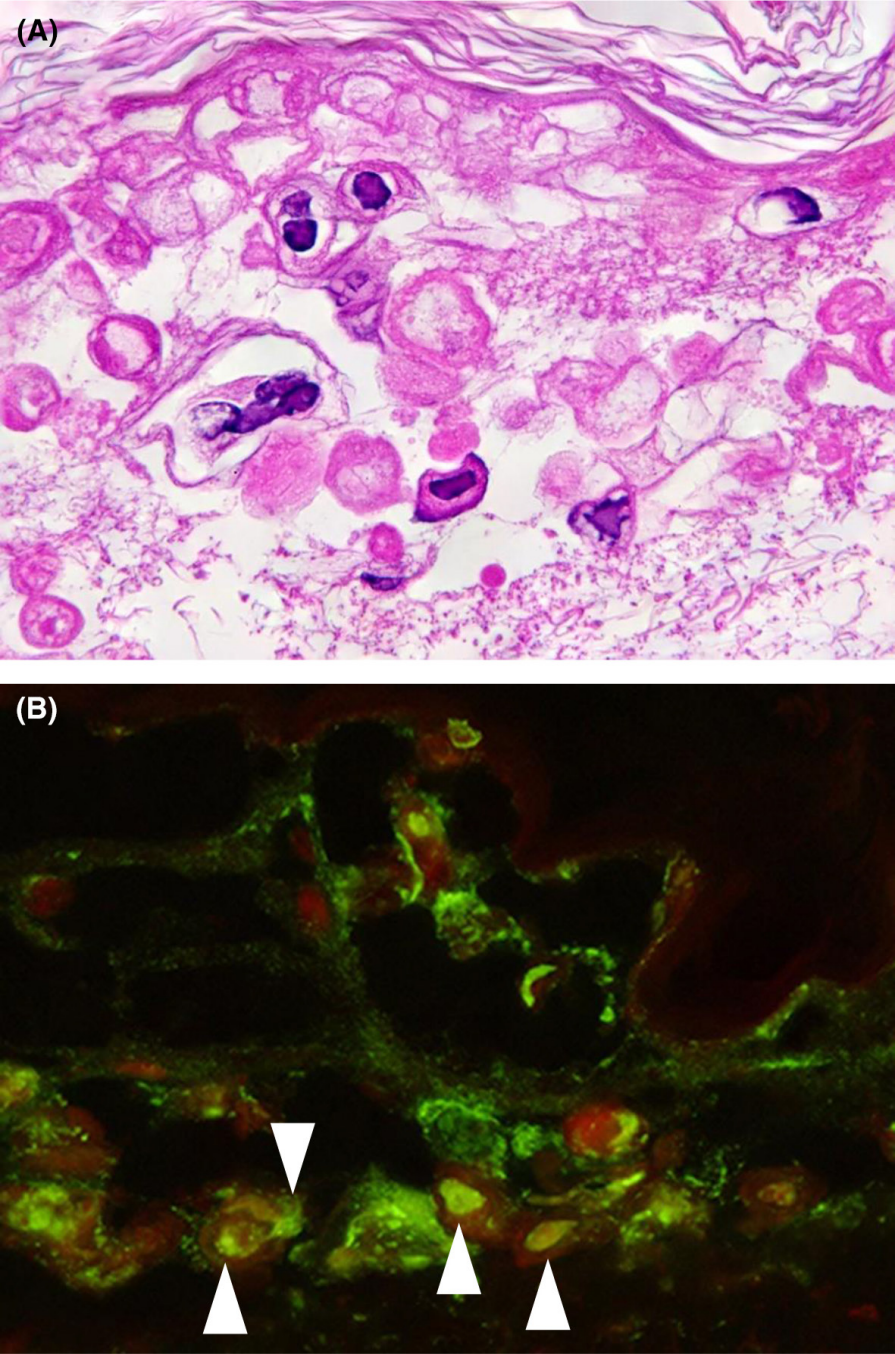
Histopathological findings of vesicles. (A) Hematoxylin–eosin stain, ×400. Intraepidermal blisters with large multinucleated giant cells and intranuclear inclusion bodies shown as a pale amorphous substance are evident. (B) Direct immunofluorescence, ×1000. The nuclei of cells in vesicles are reactive to an anti‐VZV antibody (Denka, Tokyo, Japan), as indicated by white arrowheads.

A histopathological examination revealed intraepidermal blisters with large multinucleated giant cells and intranuclear inclusion bodies (Figure 3A). There were no vascular changes in the dermis, such as endothelial swelling suggestive of CMV infection, the so‐called owl's eye sign. An immunohistochemical examination of vesicle tissue showed VZV antigens in keratinocytes on the adjacent side of blisters (Figure 3B), but failed to detect CMV antigens. Based on these findings, we diagnosed the patient with adult varicella due to primary VZV infection, CMV reactivation, and pneumonia. We administered acyclovir (ACV) at 10 mg/kg/day and added a dose of valganciclovir (VGCV) at 1800 mg/day.

Eight days after the first visit, vesicles became crusted through the administration of ACV and ELISA for VZV‐specific IgM and IgG were 1.50 and 52.6, respectively. Fourteen days later, ELISA for VZV‐specific IgM and IgG were 1.25 and 1080, respectively. Eighteen days later, the attenuation of dyspnea was noted and then oxygen inhalation and VGCV were stopped.

## 3 DISCUSSION

The immunocompromised patient was diagnosed with varicella caused by primary VZV infection based on the results of physical, blood, and histopathological examinations. The patient was also diagnosed with CMV reactivation based on the results of CMV antigen test in the blood. The previous studies reported that CMV pneumonia on CT is mainly detected as ground‐glass opacities^4^ and varicella pneumonia mainly as nodular lesions.^5^ Based on them, the ground‐glass opacities on CT in the present case might be suggested to represent CMV pneumonia. Therefore, we administered VGCV for the patient.

In the past 10 years, three cases of VZV‐infectious disease combined with CMV reactivation in immunocompromised adults have been reported in the English literature.^6^, ^7^, ^8^ In all the cases, varicella preceded CMV reactivation. The reactivation or reinfection of VZV was suggested as the developmental mechanism of varicella in the cases reported by Kasuya et al.^6^ and Hioki et al.,^7^ although Qi et al. did not definitively indicate the developmental mechanism of varicella.^8^ Based on these findings, a hypothesis arises that VZV‐infectious diseases might relate to CMV reactivation in immunocompromised patients.

The hypothesis regarding CMV reactivation in VZV‐infectious diseases may be supported by the following evidence: (i) VZV infection is reported to induce apoptosis of lymphocytes, resulting in decrease of lymphocyte count in the peripheral blood^1^, ^2^; (ii) decreased lymphocyte count in the peripheral blood is reported to relate to CMV reactivation.^9^ Furthermore, Ohtaki et al. showed that CMV was reactivated after an inoculation with VZV in an experiment on monkeys.^10^ If pneumonia was involved with CMV reactivation in the present case, it follows that VZV infection preceded CMV pneumonia in accordance with the previous reports.^6^, ^7^, ^8^

Three mechanisms have been proposed for the development of adult‐onset varicella: (i) the reactivation of latent VZV, (ii) reinfection by VZV of a different genotype from that of latent VZV, and (iii) primary VZV infection. In the case of varicella caused by the reactivation of latent VZV, VZV‐specific IgG potentially increases commonly from a certain level, whereas VZV‐specific IgM does not. In the case of varicella due to reinfection by VZV of a different genotype, VZV‐specific IgM potentially increases, whereas VZV‐specific IgG appears to rapidly increase commonly from a certain level at an early time point from the onset.^11^ In the case of varicella caused by primary VZV infection, VZV‐specific IgM definitely increases, whereas VZV‐specific IgG gradually becomes elevated over 2–3 weeks from a negative level. Increases in VZV‐specific IgM were followed by a gradual elevation in VZV‐specific IgG over 2 weeks from a negative level in the present case, which was compatible with primary VZV infection.

VZV‐specific IgG levels in SLE patients previously infected by VZV were previously reported to be significantly elevated regardless of total IgG levels through the significant activation of B cells to specific antigens as the pathogenesis of SLE; however, total IgG and complement factor 3 levels sometimes decrease with an increase in the disease activity of SLE.^12^ In the present case, the level of VZV‐specific IgG in the early stage of varicella was low, which indicated primary VZV infection in our patient.

There are a few limitations. First, CMV antigen‐detecting test using the C7 horseradish peroxidase method potentially shows lower sensitivity and specificity compared to PCR examination. Therefore, PCR examination should have been performed to detect CMV reactivation. Second, the cause of pneumonia has not been correctly decided. Bronchoalveolar lavage should have been performed to approach the developmental mechanisms of pneumonia.

There is still no definitive evidence that VZV‐infectious diseases induce CMV reactivation. Despite it, VZV‐infectious diseases might lead to more severe immunosuppressive condition through decrease of lymphocyte count especially for immunocompromised patients. Therefore, dermatologists need to consider other diseases including CMV reactivation with the appearance of varicella caused by any type of VZV infection, including reactivation, reinfection, and primary infection.

## AUTHOR CONTRIBUTIONS

**Hizuru Tomita:** Data curation; resources; writing – original draft. **Yoshimasa Nobeyama:** Conceptualization; writing – original draft. **Miya Morishima:** Data curation; resources. **Akihiko Asahina:** Writing – review and editing.

## FUNDING INFORMATION

The authors have no financial support in the case report.

## CONFLICT OF INTEREST STATEMENT

The authors have no conflict of interest to declare.

## ETHICS STATEMENT

This study protocol was approved by The Ethics Committee of The Jikei University School of Medicine and the patient provided written informed consent.

## CONSENT

Written informed consent was obtained from the patient to publish this report in accordance with the journal's patient consent policy.

## Data Availability

Additional data sharing is not applicable to this article due to ethical restrictions.
